# Impact of Occupational Therapy Interventions on Sexual Dysfunction in Epilepsy: A Case Report

**DOI:** 10.7759/cureus.51153

**Published:** 2023-12-27

**Authors:** Usha Kasar, Amitabh K Dwivedi, Prashant M Khandare

**Affiliations:** 1 Department of Occupational Therapy, Maharaj Vinayak Global University, Jaipur, IND; 2 Department of Occupational Therapy, JSS (Jagadguru Sri Shivarathreeshwara) Medical College, JSS Academy of Higher Education and Research, Mysuru, IND; 3 Department of Occupational Therapy, INHS (Indian Naval Hospital Ship) Asvini Early Intervention Centre, Mumbai, IND

**Keywords:** quality of life, depression, anxiety, sexual dysfunction, epilepsy

## Abstract

Epilepsy is a chronic neurological disorder characterized by recurrent seizures, necessitating lifelong medication management. One common side effect of these medications is sexual dysfunction. In this case report, a 37-year-old male epilepsy patient who was an office clerk by occupation presented at the outpatient department (OPD) of occupational therapy with the chief complaints of anxiety, depression, and sexual dysfunction primarily reporting of anorgasmia, which required longer foreplay to reach an effective erection leading to delayed ejaculation. The patient reported a nine-year history of complicated, partial, and generalized seizures for which he consulted the physician who prescribed him AED (antiepileptic drug) carbamazepine twice a day; however, the symptoms persisted, and the medication was changed to pregabalin. In addition to this, the patient was advised for occupational therapy intervention by the physician. In the occupational therapy department, the patient was assessed for various parameters that involved sexual functioning using the Changes in Sexual Functioning Questionnaire-Male (CSFQ-M), for anxiety using the Generalised Anxiety Disorder-7 (GAD-7) questionnaire, for depression using the Patient Health Questionnaire-9 (PHQ-9), and quality of life (QOL) using the Quality of Life in Epilepsy Inventory - 31 (QOLIE-31) questionnaire. As part of the intervention, occupational therapy was provided to the patient for four months, which mainly focused on three major areas: health promotion, remediation, and modification. Each of these methods was used at all levels of the intervention, as outlined by the EX-Permission, Limited Information, Specific Suggestions, and Intensive Therapy model (P-LI-SS-IT), which reflected positive results, as there was enhanced sexual functioning, reduced symptoms of depression, and anxiety, and improved quality of life. In conclusion, occupational therapists along with doctors and other practitioners should focus on addressing intimacy and sexuality within their practice for epilepsy patients demonstrating symptoms of sexual dysfunction, which will consequently impact an individual’s QOL. Additionally, screening and monitoring of sexual dysfunction should be included during the routine assessment of patients with epilepsy.

## Introduction

Epilepsy is a chronic neurological condition marked by seizure events and frequently needs to be treated with lifelong medication. This condition has an impact on both adults and children [1]. Over the past 70 years, a link has been found between sexual dysfunction and epilepsy, which is most commonly caused due to the side effects of antiepileptic drugs (AEDs). Premature or delayed ejaculation and erectile dysfunction in men and dyspareunia and reduced sexual desire in women are common symptoms of this sexual disorder [2-4]. Additionally, impotence and reduced libido have been associated with AEDs, including carbamazepine (CBZ), phenytoin, phenobarbital, and primidone [5].

Several studies have documented reproductive endocrine abnormalities in both women and men treated with enzyme-inducing AEDs [5,6]. Additionally, case reports have described anorgasmia and ejaculatory failure with CBZ and gabapentin (GBP) [5,6]. Occupational therapy is a broad field encompassing various therapies to help individuals improve their physical, mental, and emotional well-being. The goal of occupational therapy is to help people overcome the effects of disability by providing them with useful assistance to enhance their performance and satisfaction in activities of daily living, such as physical rehabilitation through the use of guided activity practice, practical pain, and fatigue management, assistance with addressing mental health issues, and assistance to minimize dependency and maximize independence [7]. Therefore, this case report aims to evaluate the effect of occupational therapy intervention on sexual dysfunction in epilepsy patients.

## Case presentation

Patient information

A 37-year-old male epilepsy patient who was an office clerk by occupation presented at the outpatient department (OPD) of occupational therapy with the chief complaints of anxiety, depression, and sexual dysfunction primarily reporting anorgasmia, which required longer foreplay to reach an effective erection leading to delayed ejaculation. The patient reported a nine-year history of complicated, partial, and generalized seizures for which he consulted the physician who prescribed him AED carbamazepine twice a day; however, the symptoms persisted, and the medication was changed to pregabalin. Additionally, the patient was advised for occupational therapy by the physician for which the patient proceeded with the occupational therapy intervention.

Clinical findings

In the occupational therapy department, the patient was assessed for sexual functioning using the Changes in Sexual Functioning Questionnaire-Male (CSFQ-M), for anxiety using the Generalised Anxiety Disorder-7 (GAD-7) questionnaire, for depression using the Patient Health Questionnaire-9 (PHQ-9) and quality of life (QOL) using Quality of Life in Epilepsy Inventory - 31 (QOLIE-31) questionnaire. The evaluated scores consisted of 35 on CSFQ-M for sexual functioning for which the cutoff value is ≤ 47 [8], 16 on GAD-7 for anxiety whose score ranges from 0-21 with higher scores indicating more severe GAD symptoms [9], 11 on PHQ-9 for depression, and 35.44 on QOLIE-31 for quality of life. PHQ-9 scores of 5, 10, 15, and 20 represent mild, moderate, moderately severe, and severe depression, respectively [10]. QOLIE-31 ranges from 0-100 in which more scoring represents improving quality of life and low scores represent reduced quality of life [11]. The findings reported reduced sexual functioning and QOL along with increased symptoms of depression and anxiety.

Therapeutic intervention

The occupational therapy intervention was performed in the evening, which extended from 30-45 minutes, for one session per day, six days per week for four months, and mainly focused on three major areas consisting of health promotion, remediation, and modification. The data were recorded by the principal investigator. Each of these methods was used at all levels of the intervention, as outlined by the EX-Permission, Limited Information, Specific Suggestions, and Intensive Therapy model (P-LI-SS-IT) [12-15]. The Ex-PLISSIT model is an extension of the much-used PLISSIT model. A four-step process of integrating sexuality into the occupational therapy practice was included, which involved having a conversation about sexuality, completing an occupational therapy assessment regarding sexuality, intervention planning, and application within occupational therapy, and further, providing a referral to intensive therapies was followed. The permission-giving stage, which was the initial step in attending to the patient's sexual health issues, involved the evaluation process in which the patient was allowed to express their concerns about sexual health. The practice newsletter and waiting area locations promoted the services offered and reassured the patient of confidentiality. Another way included was practice through leaflets and posters displayed in the waiting area. The next stage involved a limited information stage in which information about the impact of illness on sexuality and the effects of treatment on sexual function was explained. This stage was followed by a specific suggestion stage, which consisted of addressing of specific problem of anorgasmia, and delayed ejaculation, which was followed by the last stage of intensive therapy [12-15]. 

Health promotion involves stress-relieving activities, educational information in the form of handouts, reference material in the form of print or electronic media, and support groups. Health remediation included restoring skills, like effective communication and social engagement, as part of meeting sexual needs. Modification involved adapting or changing the environment or routine to allow for sexual activity. Following this, the patient was given Jacobson’s relaxation technique to reduce stress [16]. Additionally, energy conservation techniques and sexual positions for better participation and less energy expenditure were advised. The patient's progress was evaluated at the end of the four months using the CSFQ, GAD, PHQ-9, and QOLIE-31 questionnaires, and the observed changes were recorded.

Follow-up and outcome measures

The patient in total completed four months of sessions after which positive outcomes were demonstrated from the treatment, as there was enhanced sexual functioning, reduced symptoms of depression, and anxiety, and improved QOL as shown in Table 1.

**Table 1:** Pre-and post-comparison of outcome measures CSFQ-M = Changes in Sexual Functioning Questionnaire-Male, GAD-7 = Generalised Anxiety Disorder-7, PHQ-9 = Patient Health Questionnaire-9, QOLIE-31 = Quality of Life in Epilepsy Inventory-31

Sr. No.	Outcome Measures	Pre-treatment values	Post-treatment values after four months
1.	CSFQ-M	35	69
2.	GAD-7	16	3
3.	PHQ-9	11	2
4.	QOLIE-31	35.44	59.9

## Discussion

The report highlights a case of a 37-year-old male epilepsy patient with sexual dysfunction. The pre-intervention results showed decreased sexual dysfunction and QOL and increased depression and anxiety. After an effective occupational therapy intervention, the patient reported enhanced sexual functioning, decreased levels of depression and anxiety, and improved QOL.

Epilepsy may have an impact on sexual function, which is most likely the result of a complex etiology that includes endocrine, iatrogenic, neurological, drug-related, psychiatric, and psychosocial variables [17,18]. According to several research, hypothalamic and pituitary feedback processes have a role in the impairment of sexual function in epileptic patients. The propagation of epileptiform discharges may impact the pulsatile release of reproductive and dopamine endocrine hormones through amygdalo-hypothalamic pathways [19]. This illustrates that sexual dysfunction, hyperprolactinemia, and hypogonadism may all be caused by epilepsy. Moreover, there is evidence that suggests the use of AEDs may contribute to sexual dysfunction. Sexual dysfunction may arise from the use of traditional AEDs, such as oxcarbazepine, valproate, and carbamazepine, which can lower free testosterone, increase sex hormone-binding globulin, and accelerate the metabolism of sex hormones [2].

Depression related to seizures has also been associated with sexual dysfunction. Sexual unattractiveness is caused by low self-esteem or the worry that engaging in sexual activity will trigger a seizure [20]. Due to the prevalent involvement of neural regions in epileptic patients, anxiety problems are more likely to develop in these people. Most AEDs have several pharmacologic targets, which influence both efficacy and side effects. Sexual dysfunction is one of the significant side effects of AED medication, making it a therapy consisting of major risks [21].

The Occupational Therapy Practice Framework recognizes sexual activity as an essential component of daily living. Therefore, it falls well within the scope of practice for practitioners to initiate conversations about intimacy and sexuality. However, in clinical settings, occupational therapy (OT) practitioners often overlook this topic [22]. There are several challenges that OT practitioners face when addressing intimacy and sexuality with their clients, such as cultural and religious barriers, personal discomfort, fear of offending clients, biases toward disability and sexuality, perceived lack of knowledge, and attitudes. To overcome these hurdles, it is crucial for OT practitioners to have access to educational opportunities [23].

## Conclusions

This case report highlighted the positive outcome of occupational therapy intervention, as it resulted in enhanced sexual functioning, reduced anxiety and depression levels, and improved quality of life. The PLISSIT model creates easy-to-follow guidelines on addressing sexuality within one’s practice. It is a four-step process of integrating sexuality into the occupational therapy practice, which involves having a conversation about sexuality, completing an occupational therapy assessment regarding sexuality, intervention planning and application within occupational therapy, and further, providing a referral to intensive therapies. Occupational therapists can begin to address sexuality within their practice by creating the space to talk about it. The inclusion of sexuality within occupational therapy services will meaningfully impact an individual’s QOL. Additionally, epilepsy patients frequently do not self-report this issue due to the shame associated with it. Improving sexual functioning in epilepsy patients involves a diverse and interdisciplinary treatment. As a result, doctors and neurologists need to be on a high index of suspicion for this issue. Furthermore, the standard epilepsy workup should include screening and monitoring for sexual dysfunction.
